# Global transcriptional response to carbonic anhydrase IX deficiency in the mouse stomach

**DOI:** 10.1186/1471-2164-11-397

**Published:** 2010-06-23

**Authors:** Heini Kallio, Mika Hilvo, Alejandra Rodriguez, Eeva-Helena Lappalainen, Anna-Maria Lappalainen, Seppo Parkkila

**Affiliations:** 1 Institute of Medical Technology and School of Medicine, University of Tampere, Biokatu 6, FI-33520 Tampere, Finland; 2 Center for Laboratory Medicine, Tampere University Hospital, Biokatu 4, FI-33521 Tampere, Finland; 3 VTT Technical Research Centre of Finland, Tietotie 2, P.O. Box 1000, FI-02044 VTT, Espoo, Finland

## Abstract

**Background:**

Carbonic anhydrases (CAs) are a family of enzymes that regulate pH homeostasis in various tissues. CA IX is an exceptional member of this family because in addition to the basic CA function, it has been implicated in several other physiological and pathological processes. Functions suggested for CA IX include roles in cell adhesion and malignant cell invasion. In addition, CA IX likely regulates cell proliferation and differentiation, which was demonstrated in *Car9*^-/- ^mice. These mice had gastric pit cell hyperplasia and depletion of chief cells; however, the specific molecular mechanisms behind the observed phenotypes remain unknown. Therefore, we wanted to study the effect of CA IX deficiency on whole-genome gene expression in gastric mucosa. This was done using Illumina Sentrix^®^Mouse-6 Expression BeadChip arrays. The expression of several genes with notable fold change values was confirmed by QRT-PCR.

**Results:**

CA IX deficiency caused the induction of 86 genes and repression of 46 genes in the gastric mucosa. There was 92.9% concordance between the results obtained by microarray analysis and QRT-PCR. The differentially expressed genes included those involved in developmental processes and cell differentiation. In addition, CA IX deficiency altered the expression of genes responsible for immune responses and downregulated the expression of several digestive enzymes.

**Conclusions:**

Microarray analysis identified several potential genes whose altered expression could explain the disturbed cell lineage phenotype in the *Car9*^-/- ^gastric mucosa. The results also indicated a novel role for CA IX in the regulation of immunologic processes and digestion. These findings reinforce the concept that the main role of CA IX is not the regulation of pH in the stomach mucosa. Instead, it is needed for proper function of several physiological processes.

## Background

The carbonic anhydrases (CAs) are a family of zinc-containing metalloenzymes that catalyze the reversible hydration of carbon dioxide in the reaction: CO_2 _+ H_2_O ↔ H^+ ^+ HCO_3_^-^. They participate in several physiological processes, such as acid-base balance, CO_2 _and HCO_3_^- ^transport, respiration, bone resorption, ureagenesis, gluconeogenesis, lipogenesis, production of body fluids, and fertilization [1,2]. The CA family consists of 13 active isozymes in mammals, 12 of which are expressed and function in humans [3]. The CA isozymes have diverse tissue expression patterns, characteristic subcellular localizations, as well as unique kinetic and inhibitory properties.

CA IX is a dimeric protein associated with the cell membrane [4,5]. In its mature form, CA IX is composed of an N-terminal proteoglycan (PG) domain, a CA catalytic domain, a transmembrane region, and a short intracytoplasmic tail at the C-terminus [6]. CA IX is the only member of the CA family containing a PG domain in addition to the CA domain. Consequently, CA IX has been suggested to participate in cell adhesion processes. In fact, using MDCK (Madin-Darby canine kidney) epithelial cells, it was shown that CA IX reduces E-cadherin-mediated cell-cell adhesion by interacting with β-catenin [7].

CA IX is expressed in only few normal tissues with the expression being strongest in human, rat, and mouse gastric mucosa, where it is present from the gastric pits to the deep gastric glands [8,9]. CA IX is confined to the basolateral surface of epithelial cells and is produced by all major cell types of the gastric epithelium [8]. CA IX is an exceptional member of the CA family because it is expressed in several cancers that arise from CA IX negative tissues including renal, lung, cervical, ovarian, esophageal, and breast carcinomas [6]. However, gastric cancer and premalignant lesions have shown decreased expression of CA IX [10]. In tumor tissues, CA IX is linked with the hypoxic phenotype mediated by the hypoxia-inducible transcription factor 1 (HIF-1), which binds to the hypoxia responsive element, HRE, of the *CA9 *promoter [11]. In hypoxic conditions, cancer cells are dependent mainly on anaerobic metabolism in their energy production. This anaerobic tumor metabolism generates excesses of acidic products, such as lactic acid and H^+ ^that have to be extruded from the cell interior. It has been shown that CA IX can contribute to the acidification of the hypoxic extracellular milieu, thus helping tumor cells to neutralize the intracellular pH [12]. Accordingly, CA IX overexpression often indicates poor prognosis and resistance to classical radio- and chemotherapies [13]. However, CA IX is not only confined to the hypoxic regions of the tumors, indicating that there might be some other pathways that regulate its expression. In fact, the expression of CA IX can be induced under normoxic conditions by high cell density, and this regulation is mediated by phosphatidylinositol 3-kinase (PI3K) signaling [14]. Moreover, the mitogen-activated protein kinase (MAPK) pathway is involved in the regulation of CA IX expression via control of the *CA9 *promoter by both HIF-1-dependent and -independent signals [15]. This pathway is also interrelated with the PI3K pathway and the SP1 (specificity protein 1) transcription factor.

The generation of *Car9*^-/- ^mice has revealed that in addition to pH regulation and cell adhesion, CA IX possesses other functional roles [16]. These *Car9 *knockout mice showed no abnormalities in growth, reproductive potential, and life span. However, CA IX deficiency resulted in hyperplasia of the gastric mucosa. The hyperplastic mucosa contained an increased number and proportion of the mucus-producing pit cells whereas the number and proportion of chief cells was reduced. The total number of parietal cells remained unchanged, and accordingly, CA IX-deficient mice had normal gastric pH, acid secretion, and serum gastrin levels. Thus, these findings demonstrate that CA IX contributes to the control of differentiation and proliferation of epithelial cell lineages in the gastric mucosa. A previous study examined whether the effects of CA IX deficiency could be modified by a high-salt diet, a known co-factor of carcinogenesis [17]. These results showed that the high-salt diet slightly increased the glandular atrophy in the body mucosa in *Car9*^-/- ^C57BL/6 mice, whereas this effect was not observed in BALB/c mice. However, no metaplastic or dysplastic abnormalities were seen in the gastrointestinal epithelium of CA IX-deficient C57BL/6 mice. Thus, these observations suggest that CA IX deficiency alone may not be a significant carcinogenic factor but could initiate a carcinogenic process by affecting cell proliferation and/or differentiation in the gastric mucosa.

This study was designed to better understand the hyperplastic phenotype of the stomach mucosa of *Car9*^-/- ^mice because the molecular mechanisms behind it are currently unknown. In addition, we wanted to more specifically elucidate the functional roles of CA IX, as there is a growing body of evidence showing that they extend far beyond pH regulation. For this purpose, a genome-wide expression analysis was performed. Microarray data analysis revealed several genes whose expression was changed due to *Car9 *gene disruption in the gastric mucosa.

## Results

### Transcriptional response to Car9 deficiency in the stomach wall

Stomach RNA from 6 *Car9*^-/- ^mice and 6 wild-type mice was analyzed by microarray. The analysis revealed 86 upregulated genes and 46 downregulated genes, using a fold change cut-off of ± 1.4 for up- and downregulated expression, respectively (See additional file 1: List of genes differentially expressed in the stomach of *Car9*^-/- ^mice). This cut-off value has been proposed as an adequate level above which there is a high correlation between microarray and QRT-PCR data, regardless of other factors such as spot intensity and cycle threshold [18]. The fold changes ranged from 10.46 to -12.14. Herein, a list of genes using a cut-off value of ± 2.5-fold is shown (Tables 1 and 2). When using these criteria, all the genes with significantly (p < 0.05) altered expression are displayed, that is, 14 genes with induced expression and 21 genes with repressed expression. The list of all the regulated genes was functionally annotated (see additional file 2: Functional annotation of genes regulated in the stomach of *Car9 *knockout mice), showing enrichment of hydrolase activity, developmental processes, cell differentiation, proteolysis, peptidase activity, structural molecule activity, and immune system process, among others. The functional annotation categories and gene numbers in each category are shown in Table 3.

**Table 1:** Genes with upregulated expression using a cut-off value of 2.5-fold.

Gene symbol	Description	GenBank number	FC	QRT-PCR
Cym	chymosin	NM_001111143	10.46	19.66

Slc9a3	solute carrier family 9 (sodium/hydrogen exchanger), member 3	NM_001081060	8.07	10.72

U46068	cDNA sequence U46068, transcript variant 2	NM_153418	5.95	

Dmbt1	deleted in malignant brain tumors 1	NM_007769	5.68	5.29

Il1rl1	interleukin 1 receptor-like 1, transcript variant 2	NM_010743	5.38	8.95

Tm4sf5	transmembrane 4 superfamily member 5	NM_029360	4.26	

9130204L05Rik	RIKEN cDNA 9130204L05 gene	NM_001101461	4.19	

Sftpd	surfactant associated protein D	NM_009160	4.11	3.69

Nccrp1	non-specific cytotoxic cell receptor protein 1 homolog (zebrafish)	NM_001081115	3.81	

Pkp4	plakophilin 4, transcript variant 1	NM_026361	3.54	1.09

Sprr2d	small proline-rich protein 2D	NM_011470	3.46	

Gm14446	predicted gene 14446, transcript variant 2	NM_001101605	3.45	

Sprr3	small proline-rich protein 3	NM_011478	3.39	

Sprr2i	small proline-rich protein 2I	NM_011475	3.31	

Sprr1a	small proline-rich protein 1A	NM_009264	3.30	

Ivl	involucrin	NM_008412	3.08	

Serpinb12	serine (or cysteine) peptidase inhibitor, clade B (ovalbumin), member 12	NM_027971	3.02	

Krt10	keratin 10	NM_010660	3.01	

Gm94	predicted gene 94	NM_001033280	2.94	

Krt13	keratin 13	NM_010662	2.94	

Gsdmc2	gasdermin C2, transcript variant 2	NM_177912	2.88	

Lor	loricrin	NM_008508	2.84	

Dmkn	dermokine, transcript variant 2	NM_172899	2.78	

Ly6d	lymphocyte antigen 6 complex, locus D	NM_010742	2.69	

Krt1	keratin 1	NM_008473	2.52	

**Table 2:** Genes with downregulated expression using a cut-off value of -2.5-fold.

Gene symbol	Description	GenBank number	FC	QRT-PCR
Try4	trypsin 4	NM_011646	-12.14	

Prss1	protease, serine, 1 (trypsin 1)	NM_053243	-10.57	

Amy2a5	amylase 2a5, pancreatic	NM_001042711	-9.85	

Cela2a	chymotrypsin-like elastase family, member 2A	NM_007919	-7.59	-16.23

Gm12888	predicted gene 12888	NM_001033791	-7.12	

Gdf9	growth differentiation factor 9	NM_008110	-7.04	

Blm	bloom syndrome homolog (human), transcript variant 1	NM_007550	-6.27	

Pnlip	pancreatic lipase	NM_026925	-6.23	-23.88

Cfd	complement factor D (adipsin)	NM_013459	-5.35	-27.45

Ctrb1	chymotrypsinogen B1	NM_025583	-5.00	

Mug1	murinoglobulin 1	NM_008645	-4.30	

Sostdc1	sclerostin domain containing 1	NM_025312	-4.17	

Tmed6	transmembrane emp24 protein transport domain containing 6	NM_025458	-4.07	

Cpa1	carboxypeptidase A1	NM_025350	-3.95	

Slc27a2	solute carrier family 27 (fatty acid transporter), member 2	NM_011978	-3.80	

Adipoq	adiponectin, C1Q and collagen domain containing	NM_009605	-3.74	-20.51

Car3	carbonic anhydrase 3	NM_007606	-3.72	-15.39

Egf	epidermal growth factor	NM_010113	-3.68	-3.71

Abpg	androgen binding protein gamma	NM_178308	-3.67	

Slc38a5	solute carrier family 38, member 5	NM_172479	-3.64	

Chia	chitinase, acidic	NM_023186	-3.52	

LOC638418	PREDICTED: similar to Ela3 protein	XM_914439	-3.48	

LOC100043836	PREDICTED: similar to lacrimal androgen-binding protein delta, transcript variant 1	XM_001481113	-3.41	

EG640530	PREDICTED: predicted gene, EG640530	XM_917532	-3.37	

Nrn1	neuritin 1	NM_153529	-3.36	

Cela3b	chymotrypsin-like elastase family, member 3B	NM_026419	-3.32	-75.74

Spink3	serine peptidase inhibitor, Kazal type 3	NM_009258	-3.32	

Scd1	stearoyl-Coenzyme A desaturase 1	NM_009127	-3.27	

Ctrl	chymotrypsin-like	NM_023182	-3.19	

Gper	G protein-coupled estrogen receptor 1	NM_029771	-2.96	

Sycn	syncollin	NM_026716	-2.91	

Bhlha15	basic helix-loop-helix family, member a15	NM_010800	-2.77	

Cpb1	carboxypeptidase B1 (tissue)	NM_029706	-2.73	-18.22

Slc5a8	solute carrier family 5 (iodide transporter), member 8	NM_145423	-2.68	

Cyp2e1	cytochrome P450, family 2, subfamily e, polypeptide 1	NM_021282	-2.65	

Zg16	zymogen granule protein 16	NM_026918	-2.57	

**Table 3:** The functional annotation categories for the genes regulated by CA IX deficiency.

Functional category	Total regulated genes	Upregulated genes	Downregulated genes
Developmental processes*	20	13	7

Keratinization and keratinocyte differentiation	8	8	0

Structural molecule activity	13	13	0

Embryo implantation, female pregnancy, and menstrual cycle	3	3	0

Rhythmic process	4	3	1

Multi-organism process	5	5	0

Cell differentiation	16	12	4

Serine-type endopeptidase activity, serine hydrolase activity, and serine-type peptidase activity	9	3	6

Proteolysis	15	6	9

Peptidase activity	14	5	9

Endopeptidase activity	10	4	6

Trypsin and chymotrypsin activity	4	1	3

Hydrolase activity	22	8	14

Structural constituent of cytoskeleton	5	5	0

Cell communication	4	4	0

Metallocarboxypeptidase activity, carboxypeptidase activity, and metalloexopeptidase activity	3	0	3

Serine-type endopeptidase inhibitor activity, endopeptidase inhibitor activity, and protease inhibitor activity	4	2	2

Taxis and chemotaxis	4	3	1

Response to external stimulus	7	4	3

Immune system process	10	5	5

Defense response	7	5	2

Positive regulation of cell differentiation	3	2	1

PPAR signaling pathway	3	0	3

Leukocyte migration	3	2	1

*A representative category for the following overlapping categories: epidermis morphogenesis, tissue morphogenesis, epidermis development, ectoderm development, tissue development, organ development, anatomical structure development, anatomical structure morphogenesis, cellular developmental process, multicellular organismal development, and epidermal cell differentiation.

### Confirmation of microarray results by QRT-PCR

**Figure 1:**
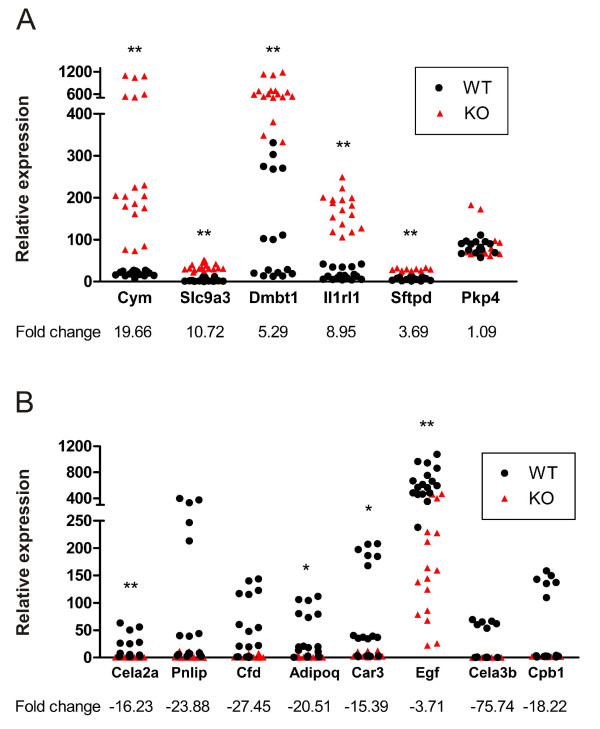
**Verification of microarray data from Car9^-/- ^mice by QRT-PCR**. The expression of various transcripts in 6 *Car9*^-/- ^mice was compared to that in 6 wild-type controls. The normalized values of triplicate experiments are shown. A, QRT-PCR analysis of 6 genes with induced expression. B, QRT-PCR evaluation of 8 genes with repressed expression. Statistically significant differences relative to wild-type mice were determined. *p < 0.05; **p < 0.01.

Changes in the expression levels of selected genes were confirmed, and microarray results were validated by QRT-PCR using the same RNA samples as those used for the microarray. Fourteen genes with notable fold change values were selected for validation based on the results of functional annotation. The selected genes involved representatives from different functional categories. Thirteen (92.9%) out of 14 genes showed concordant results between microarray analysis and QRT-PCR (Tables 1 and 2, Figure 1). The sole discrepant result was *Pkp4*, which was upregulated according to the microarray and did not change according to the QRT-PCR (1.09-fold).

## Discussion

A previous study showed a hyperplastic phenotype of gastric body mucosa in *Car9*^-/- ^mice [16]. CA IX deficiency led to overproduction of gastric pit cells and reduction of chief cells. Quite surprisingly, the gastric pH of CA IX-deficient mice remained unaltered. Based on these observations, it can be concluded that the main role of CA IX in the stomach mucosa is not related to pH regulation but it is required for normal gastric morphogenesis and homeostasis within the gastric mucosa. In this paper, we report alterations in mRNA expression that might contribute to the phenotypic changes reported in the stomach of *Car9*-deficient mice. However, one must take into consideration that some of these changes can merely reflect differences in the relative proportions of various cell types within the gastric mucosa.

As expected, the *Car9*^-/- ^phenotype changed the expression of genes involved in developmental processes and cell differentiation. The depletion of chief cells in *Car9*^-/- ^gastric mucosa may be explained by the significant downregulation of the *basic helix-loop-helix family, member a15/Mist1 *(*Bhlha15*) gene, which is a class B basic helix-loop-helix (bHLH) transcription factor that exhibits acinar cell-specific expression [19]. Mist1 gene expression is observed in a wide array of tissues with serous type secretion including the pancreas, salivary glands, chief cells of the stomach, Paneth cells of the small intestine, seminal vesicles, and lacrimal glands [20]. The deletion of *Mist1 *blocks normal mucous neck cell redifferentiation into zymogenic chief cells as all basal zymogen-secreting cells in *Mist1*^-/- ^mice show multiple structural defects [21].

Another interesting candidate with regard to cell lineage disturbance in the gastric epithelium of CA IX-deficient mice is *epidermal growth factor *(*Egf*), which has an expression that was significantly decreased when compared to wild-type mice. Among growth factors, the EGF family includes important agents for gastric mucosa. EGF is a single-chain polypeptide of 53 amino acid residues, which is found mainly in the submandibular glands and Brunner's glands of the gastrointestinal tract [22]. EGF binds and activates the epidermal growth factor receptor resulting in cellular proliferation, differentiation, and survival [23]. Interestingly, several investigators have reported that EGF receptors are enriched in rodent chief cells [24,25], suggesting the importance of EGF function in these cells. Therefore alterations in the expression of EGF receptor ligands or, in this case, EGF could contribute to the differentiation and function of chief cells.

One of the most strongly upregulated genes of this study was *deleted in malignant brain tumors 1 *(*Dmbt1*), which belongs to the scavenger receptor cysteine-rich (SRCR) superfamily of proteins. This is a superfamily of secreted and membrane-bound proteins with SRCR domains that are highly conserved down to sponges [26,27]. The expression of DMBT1 is the highest in epithelia and is usually observed on the apical cell surface or in luminal exocrine secretions. In mice, DMBT1 is most strongly expressed in the gastrointestinal system [28]. DMBT1 is proposed to be a tumor suppressor [26,29,30] and/or a regulator of epithelial differentiation [31], as well as having roles in innate immune defense and inflammation [32,33]. DMBT1 is located in the crypt cells of the human, mouse, and rabbit small intestine [31,34,35] and the neck region of normal human gastric mucosa [36], which are predominantly composed of stem/progenitor cells. Thus, this gene is potentially involved in the physiological renewing process of gastrointestinal epithelia. A role for DMBT1 in innate immune defense has been demonstrated in various studies. The human DMBT1 glycoprotein, expressed in salivary glands, airways, and genital tract, binds various bacterial pathogens and viruses [37-40]. Additionally, expression of mouse DMBT1 in the gastrointestinal tract is regulated by bacteria [41,42], and its expression is increased during infection [43]. It has also been shown that there is an association of a *Dmbt1 *variant allele with Crohn's disease and there is a correlation of expression levels of *Dmbt1 *with inflammatory bowel disease severity [44].

Furthermore, DMBT1 binds surfactant proteins SP-D and SP-A. These are collagen- containing, (C-type) calcium-dependent lectins called collectins which interact with glycoconjugates and lipids on the surface of microorganisms mostly through their carbohydrate recognition domains (CRDs) [45]. SP-D and SP-A are involved in a range of immune functions, including viral neutralization, aggregation and killing of bacteria and fungi, and clearance of apoptotic and necrotic cells. In immunologically naïve lungs, they downregulate inflammatory reactions, but when challenged with LPS or apoptotic cells they induce phagocytosis by macrophages, pro-inflammatory cytokine production, and enhancement of adaptive immune responses [46]. It is interesting that in the present study, in addition to *Dmbt1*, the expression of *surfactant associated protein D *(*Sftpd or SP-D*) was highly induced in *Car9*^-/- ^mice. Thus, the upregulation of both *Dmbt1 *and *Sftpd *suggest that CA IX deficiency induced an immune process in the gastric mucosa. It should also be noted that CA IX has been suggested to bind to dendritic cells in a receptor-mediated manner and scavenger receptors appear to play an important role in this process [47]. In addition, CA IX seems to activate an immune response by a mechanism characteristic to heat shock proteins. Thus, CA IX could directly interact with DMBT1 via a scavenger receptor.

In fact, the disruption of *Car9 *gene caused disregulation of several genes that are involved in immune processes. This corroborates the results from a previous study where gastric submucosal inflammation was detected in the body region in a majority of C57BL/6 *Car9*^-/- ^mice [17]. In the present study, *Il1rl1/ST2 *transcript variant 2 mRNA was highly induced due to CA IX deficiency. The *Il1rl1/ST2 *gene is a member of the interleukin-1 (IL-1) receptor family and produces a soluble secreted form (sST2) and a transmembrane form (ST2L), coded by transcript variants 2 and 1, respectively. The membrane-bound form is expressed primarily by hematopoietic cells and the soluble form is predominantly expressed by fibroblasts [48]. In particular, ST2L is preferentially expressed in murine and human Th2 cells and can be used as a specific marker of Th2 cells in *in vitro *experiments [49-52]. Therefore, the function of ST2L has been suggested to correlate with Th2 cell-mediated immunological responses and IL-33, a newly discovered member of the IL-1 cytokine family, has been reported as a specific ligand for ST2L [53]. Interestingly, several studies have shown that the level of soluble ST2 in sera is elevated in asthmatic disease [54,55]. Therefore, it has been suggested that soluble ST2 may also play a critical role in Th2 cell-mediated diseases. In fact, it has been demonstrated that soluble ST2 acts as an antagonistic decoy receptor for IL-33 using a murine thymoma cell line, EL-4, which stably expresses ST2L, and a murine model of asthma [56]. This suggests that soluble ST2 negatively modulates the production of Th2 cytokines through IL-33 signaling in allergic airway inflammation. It is interesting to note that in the present study, the mRNA level of IL-33 was also elevated ~ 2-fold, although this change was not statistically significant. Thus, it seems plausible that CA IX is somehow involved in regulation of the Th2 response.

CA IX could also contribute to the development of the immune system as CA IX deficiency significantly downregulated the *bloom syndrome homolog (human) *gene (*Blm*), which encodes an ATP-dependent DNA helicase that belongs to an evolutionarily conserved family of RecQ helicases [57]. Mutation of *Blm *causes a rare human autosomal recessive disorder called Bloom's syndrome (BS), which is characterized by marked genomic instability. BS is associated with growth retardation, profound susceptibility to most cancer types, and immunodeficiency [58], with the latter two features accounting for early death [59]. The functions of BLM have been only partially characterized. However, it has been shown that BLM has a role in the proliferation and survival of both developing and mature T lymphocytes, and its deletion leads to defective T-cell immunity [60]. Additionally, the conditional knockout of *Blm *contributed to compromised B cell development and maintenance, strongly impaired T cell-dependent and -independent antibody responses after immunization, and a propensity for developing B cell lymphomas [61]. Therefore, it is conceivable that *Car9*^-/- ^mice have compromised acquired immunity responses.

An unexpected finding was that several *small proline-rich proteins *(*Sprr*) were notably upregulated including *Sprr1a*, *Sprr2d*, *Sprr2e*, *Sprr2i*, and *Sprr3*. However, only the induction of *Sprr1a *was statistically significant. SPRR proteins were originally identified as markers for terminal squamous cell differentiation where they are precursors of the cornified envelope [62]. Additionally, SPRR proteins are expressed in various nonsquamous tissues, and their biological function is not restricted to structural proteins of the cornified envelope [63]. It has been shown that SPRR proteins participate in the response to various stresses in many tissues without a stratified epithelium. In the biliary tract, SPRR2 members appear to contribute to modification of the biliary barrier under conditions of stress [64]. Likewise, SPRR1A has been identified as a novel stress-inducible downstream mediator of gp130 cytokines in cardiomyocytes with a cardioprotective effect against ischemic stress [65]. Furthermore, in mice, SPRR2A protein was reported to be highly induced in gastric mucosa infected by *Helicobacter heilmannii *[66]. In addition, it has been suggested that SPRR1A is a regeneration-associated protein because its induction in neuronal injury correlates with regenerative capacity, with virtually all injured dorsal root ganglion neurons expressing SPRR1A one week after sciatic nerve injury [67]. Therefore, SPRR genes are presumably induced in response to different stress conditions and contribute to cell protection, tissue remodeling, and/or tissue regeneration. In the light of these findings it seems plausible that CA IX deficiency creates a stress condition in the gastric mucosa, which leads to upregulation of some protective factors.

Our analysis identified mRNAs from four members of solute carrier family proteins to be misregulated in the *Car9*^-/- ^mice stomach mucosa. These solute carrier proteins are involved in membrane transport of various molecules. The expression of *Slc9a3 *was strongly induced, whereas the expression of *Slc27a2*, *Slc38a5*, and *Slc5a8 *was repressed. The basic function of SLC9A3 or NHE3 is the exchange of extracellular Na^+ ^for intracellular H^+^, thus causing either a rise in intracellular pH or, if coupled to the action of other transporters, an increase in cell volume [68]. In the gastrointestinal tract, SLC9A3 is expressed in the apical membrane and recycling endosomes of the ileum, jejunum, colon, and stomach [69]. Further studies are needed to elucidate the possible interplay between CA IX and SLC9A3 in the gastric mucosa.

Surprisingly, among the most downregulated genes we found several genes involved in digestion. These included *trypsin 1*, *amylase 2a5*, *chymotrypsin-like elastase family member 2A*, and *pancreatic lipase *with statistically significant p-values and others, such as *chymotrypsinogen B1*, *carboxypeptidase A1*, and *carboxypeptidase B1 *with nonsignificant p-values. The implication of this finding remains unclear. A possible explanation for this might be that downregulation of these enzymes is a secondary event. If it is assumed that these enzymes are produced by chief cells, the reduced number of chief cells in the *Car9*^-/- ^mice gastric mucosa can also cause a decrease in the amount of the enzymes they produce. However, the exact mechanism behind this phenomenon remains to be elucidated.

## Conclusions

In conclusion, CA IX deficiency was shown to alter the expression of various genes in the gastric mucosa. The number of affected genes and the magnitude of changes were relatively high, indicating that CA IX has a remarkable role in gastric functions. Microarray analysis revealed several genes involved in developmental processes and cell differentiation, which could explain the cell lineage disturbance in the gastric epithelium of *Car9*^-/- ^mice. Interestingly, some of the regulated genes identified in this study are involved in digestion as well as functions of the immune and defense responses. This finding suggests that CA IX deficiency compromises the immune system in the gastric epithelium implying yet another role for this multifunctional enzyme.

## Methods

### Animal model and tissue preparation

Generation of the targeted disruption of the *Car9 *gene has been previously described by Ortova Gut et al. [16]. These *Car9-*deficient C57BL/6 mice were produced and maintained in the animal facility of the University of Oulu and then delivered to the animal facility of the University of Tampere. Permissions for use of the experimental mice were obtained from the Animal Care Committees of the Universities of Oulu and Tampere. Six *Car9*^-/- ^mice (3 males and 3 females) and six wild-type control mice (3 males and 3 females) were kept under tightly controlled specific pathogen-free conditions and fed identical diets. The mice were sacrificed at approximately 11 (SD = ± 1.35) months of age. Tissue specimens from the body of the stomach were immediately collected, immersed in RNAlater solution (Ambion, Austin, TX, USA), and frozen at -80°C.

### RNA isolation

Total RNA was obtained using the RNeasy RNA isolation kit (Qiagen, Valencia, USA) following the manufacturer's instructions. Residual DNA was removed from the samples using RNase-free DNase (Qiagen). The RNA concentration and purity were determined by measurement of the optical density (OD) at 260 and 280 nm. All samples had an OD260/OD280 ratio of 1.95 or higher.

### Microarray analysis

All microarray data reported in the present article are described in accordance with MIAME guidelines, have been deposited in NCBI's Gene Expression Omnibus public repository [70], and are accessible through GEO Series accession number GSE20981 [71]. Microarray experiments were performed in the Finnish DNA Microarray Centre at the Turku Centre for Biotechnology. Stomach RNA samples from 6 *Car9*^-/- ^mice were used. As controls, RNA samples from the stomach of 6 wild-type mice were used. All 12 samples were analyzed individually. 400 ng of total RNA from each sample was amplified using the Illumina^® ^TotalPrep RNA Amplification kit (Ambion) as recommended by the manufacturer. The *in vitro *transcription reaction, which was conducted for 14.5 h, included labeling of the cRNA by biotinylation.

### Hybridization and scanning

Labeled and amplified cRNAs (1.5 μg/array) were hybridized to Illumina's Sentrix^® ^Mouse-6 Expression Bead Chips (Illumina, Inc., San Diego, CA) at 58°C for 18 h according to Illumina^® ^Whole-Genome Gene Expression with IntelliHyb Seal System Manual. The arrays were washed and then stained with 1 μg/ml cyanine3-streptavidin (Amersham Biosciences, Buckinghamshire, UK). The Illumina BeadArray™ Reader was used to scan the arrays according the manufacturer's instructions. The numerical results were extracted with the Illumina's BeadStudio software without any normalization or background subtraction.

### Data analysis

Array data were normalized with Chipster (v1.3.0) using the quantile normalization method. The data were filtered according to the SD of the probes. The percentage of data that did not pass through the filter was adjusted to 99.4%, implicating a SD value of almost 3. Statistical analysis was next performed using the empirical Bayes t-test for the comparison of two groups. Finally, the probes were further filtered according to fold change with ± 1.4 as the cut-off for up- and downregulated expression, respectively. The functional annotation tool DAVID (Database for Annotation, Visualization and Integrated Discovery) [72,73] was used to identify enriched biological categories among the regulated genes when compared to all the genes present on Illumina's Sentrix Mouse-6 Expression Bead Chip. The annotation groupings analyzed were Gene Ontology biological process and molecular functions, SwissProt comments, SwissProt Protein Information Resources Keywords, Kyoto Encyclopedia of Genes and Genomes pathway, and Biocarta pathway. Results were filtered to remove categories with EASE (Expression Analysis Systematic Explorer) scores greater than 0.05. Overlapping categories with the same gene members were removed to yield a single representative category.

### Quantitative real-time PCR

For quantitative real-time PCR (QRT-PCR), the same samples were used as for the microarray analysis. 5 μg of each RNA sample was converted into first strand cDNA using a First Strand cDNA Synthesis Kit (Fermentas, Burlington, Canada) using random hexamer primers following the manufacturer's instructions. The relative expression levels of the target genes in the stomach wall were assessed by QRT-PCR using the LightCycler detection system (Roche, Rotkreuz, Switzerland). The primer sets for the target genes (Table 4) were designed using Primer3 [74], based on the complete cDNA sequences deposited in GenBank. The specificity of the primers was verified using NCBI Basic Local Alignment Search Tool (BLAST) [20]. The house-keeping genes *β-actin *(*Actb*), *hypoxanthine guanine phosphoribosyl transferase *(*Hprt*), and *succinate dehydrogenase complex, subunit A *(*Sdha*) were used as internal controls to normalize the cDNA samples for possible differences in quality and quantity (Table 4). The *Actb *and *Hprt *primers are available in the public Quantitative PCR Primer Database [75] under the identification numbers 634 and 10050, respectively. The *Sdha *primers were obtained from the public PrimerBank database [76] with the identification number 15030102a2. To avoid amplification of genomic DNA, the primers from each primer pair were located in different exons, when possible.

**Table 4:** Sequences of the QRT-PCR primers used in this study.

Gene symbol	Description	GenBank number	Forward primer (5'-3')	Reverse primer (5'-3')
Adipoq	adiponectin, C1Q and collagen domain containing	NM_009605	TCCTGCTTTGGTCCCTCCAC	TCCTTTCTCTCCCTTCTCTCC

Car3	carbonic anhydrase 3	NM_007606	GCTCTGCTAAGACCATCC	ATTGGCGAAGTCGGTAGG

Cela2a	chymotrypsin-like elastase family, member 2A	NM_007919	TGATGTGAGCAGGGTAGTTGG	CACTCGGTAGGTCTGATAGTTG

Cela3b	chymotrypsin-like elastase family, member 3B	NM_026419	TGCCTGTGGTGGACTATGAA	CAGCCCAAGGAGGACACAA

Cfd	complement factor D (adipsin)	NM_013459	AACCGGACAACCTGCAATC	CCCACGTAACCACACCTTC

Cpb1	carboxypeptidase B1 (tissue)	NM_029706	GGTTTCCACGCAAGAGAG	GTTGACCACAGGCAGAACA

Cym	chymosin	NM_001111143	ATGAGCAGGAATGAGCAG	TGACAAGCCACCACTTCACC

Dmbt1	deleted in malignant brain tumors 1	NM_007769	GCACAAGTCCTCCATCATTC	AGACAGAGCAGAGCCACAAC

Egf	epidermal growth factor	NM_010113	GCTCGGTGTTTGTGTCGTG	CTGTCCCATCATCGTCTGG

Il1rl1	interleukin 1 receptor-like 1, transcript variant 2	NM_010743	ATTCTCTCCAGCCCTTCATC	AAGCCCAAAGTCCCATTCTC

Pkp4	plakophilin 4, transcript variant 1	NM_026361	GAACATAACCAAAGGCAGAGG	GGTGGACAGAGAAGGGTGTG

Pnlip	pancreatic lipase	NM_026925	CCCGCTTTCTCCTCTACACC	TCACACTCTCCACTCGGAAC

Sftpd	surfactant associated protein D	NM_009160	CCAACAAGGAAGCAATCTGAC	TCTCCCATCCCGTCCATCAC

Slc9a3	solute carrier family 9 (sodium/hydrogen exchanger), member 3	NM_001081060	TGACTGGCGTGGATTGTGTG	ACCAAGGACAGCAGGAAGG

Actb	actin, beta	NM_007393	AGAGGGAAATCGTGCGTGAC	CAATAGTGATGACCTGGCCGT

Hprt	hypoxanthine guanine phosphoribosyl transferase	NM_013556	AGCTACTGTAATGATCAGTCAACG	AGAGGTCCTTTTCACCAGCA

Sdha	succinate dehydrogenase complex, subunit A	NM_023281	GCTTGCGAGCTGCATTTGG	CATCTCCAGTTGTCCTCTTCCA

Each PCR reaction was performed in a total volume of 20 μl containing 0.5 μl of first strand cDNA, 1x of QuantiTect SYBR Green PCR Master Mix (Qiagen, Hilden, Germany), and 0.5 μM of each primer. Amplification and detection were carried out as follows: after an initial 15 min activation step at 95°C, amplification was performed in a three-step cycling procedure for 45 cycles: denaturation at 95°C for 15 s, annealing at a temperature determined according to the T_m _for each primer pair for 20 s, and elongation at 72°C for 15 s (the ramp rate was 20°C/s for all the steps), followed by a final cooling step. Melting curve analysis was always performed after the amplification to examine the specificity of the PCR. To quantify the levels of the target transcripts, a standard curve was established for each gene using five-fold serial dilutions of known concentrations of purified PCR products generated with the same primer pairs. Every cDNA sample was tested in triplicate. The mean and SD of the 3 crossing point (Cp) values were calculated for each sample and a SD cut-off of 0.2 was set. Accordingly, when the SD of the triplicates of a sample was greater than 0.2, the most outlying replicate was excluded and the analysis was continued with the two remaining replicates. Using a specific standard curve, the Cp values were then transformed by the LightCycler software into copy numbers. The expression value for each sample was the mean of the copy numbers for the sample's replicates. The geometric mean of the three internal control genes was used as an accurate normalization factor for gene expression levels [77]. The final relative mRNA expression was given as the expression value of the sample divided by the corresponding normalization factor and multiplied by 10^3^.

### Statistical analyses

Statistical analyses of the microarray were performed using the empirical Bayes t-test for comparison of the two groups, and the p-values are shown in Additional file 1. For the QRT-PCR results, the Mann-Whitney test was used to evaluate differences in group values for *Car9*^-/- ^mice vs. wild-type mice.

## List of abbreviations used

CA IX: carbonic anhydrase IX; HIF-1: hypoxia-inducible transcription factor 1; HRE: hypoxia responsive element; MAPK: mitogen-activated protein kinase; PI3K: phosphatidylinositol 3-kinase; QRT-PCR: quantitative real-time PCR.

## Authors' contributions

HK prepared the samples, participated in the microarray data analysis and QRT-PCR confirmations, and drafted the manuscript. MH participated in the design of the study and microarray data analysis. AR participated in the experimental design of the study and critically reviewed the manuscript. EHL and AML participated in the QRT-PCR confirmations. SP conceived the study, participated in its design and coordination, and critically reviewed the manuscript. All authors read and approved the final manuscript.

## Supplementary Material

Additional file 1 **List of genes differentially expressed in the stomach of *Car9*^-/- ^mice**. The *Car9*^-/- ^and control groups contained samples from 6 mice. Microarray data were normalized with Chipster (v1.3.0) using the quantile normalization method. Statistical analysis was performed using the empirical Bayes t-test for the comparison of two groups. The probes were filtered according to fold change with cut-off of ± 1.4 for up- and down-regulated expression, respectively. Click here for file

Additional file 2 **Functional annotation of genes regulated in the stomach of *Car9 *knockout mice**. The functional annotation tool DAVID was used to identify enriched biological categories among the differentially expressed genes as compared to all genes present in Illumina's Sentrix Mouse-6 Expression Bead Chip. Click here for file
